# Effects of Cholinergic Stimulation with Pyridostigmine Bromide on Chronic Chagasic Cardiomyopathic Mice

**DOI:** 10.1155/2014/475946

**Published:** 2014-08-24

**Authors:** Marília Beatriz de Cuba, Marcus Paulo Ribeiro Machado, Thais Soares Farnesi, Angelica Cristina Alves, Livia Alves Martins, Lucas Felipe de Oliveira, Caroline Santos Capitelli, Camila Ferreira Leite, Marcos Vinícius Silva, Juliana Reis Machado, Henrique Borges Kappel, Helioswilton Sales de Campos, Luciano Paiva, Natália Lins da Silva Gomes, Ana Carolina Guimarães Faleiros, Constança Felicia de Paoli de Carvalho Britto, Wilson Savino, Otacílio Cruz Moreira, Virmondes Rodrigues Jr., Nicola Montano, Eliane Lages-Silva, Luis Eduardo Ramirez, Valdo Jose Dias da Silva

**Affiliations:** ^1^Natural and Biological Sciences Institute, Triangulo Mineiro Federal University, Praca Manoel Terra 330, Centro, 38025-015 Uberaba, MG, Brazil; ^2^Oswaldo Cruz Institute, 21040-900 Rio de Janeiro, RJ, Brazil; ^3^Department of Clinical Sciences, Internal Medicine II, L. Sacco Hospital, University of Milan, 20157 Milan, Italy

## Abstract

The aim of the present study was to assess the effects of an anticholinesterase agent, pyridostigmine bromide (Pyrido), on experimental chronic Chagas heart disease in mice. To this end, male C57BL/6J mice noninfected (control:Con) or chronically infected (5 months) with *Trypanosoma cruzi* (chagasic:Chg) were treated or not (NT) with Pyrido for one month. At the end of this period, electrocardiogram (ECG); cardiac autonomic function; heart histopathology; serum cytokines; and the presence of blood and tissue parasites by means of immunohistochemistry and PCR were assessed. In NT-Chg mice, significant changes in the electrocardiographic, autonomic, and cardiac histopathological profiles were observed confirming a chronic inflammatory response. Treatment with Pyrido in Chagasic mice caused a significant reduction of myocardial inflammatory infiltration, fibrosis, and hypertrophy, which was accompanied by a decrease in serum levels of IFN*γ* with no change in IL-10 levels, suggesting a shift of immune response toward an anti-inflammatory profile. Lower nondifferent numbers of parasite DNA copies were observed in both treated and nontreated chagasic mice. In conclusion, our findings confirm the marked neuroimmunomodulatory role played by the parasympathetic autonomic nervous system in the evolution of the inflammatory-immune response to *T. cruzi* during experimental chronic Chagas heart disease in mice.

## 1. Introduction

Chagas disease, also known as American Trypanosomiasis, an endemic parasitic disease caused by a flagellate protozoan (*Trypanosoma cruzi*), is highly prevalent throughout Latin America. Its major clinical manifestation is the chronic chagasic cardiomyopathy (CCC), which affects 1/3 of the chronically infected patients and may present severe symptoms and signs, such as congestive heart failure, thromboembolic phenomena, cardiac arrhythmias, and sudden death [1–4]. Pathological processes in the heart include mononuclear inflammatory infiltration, focal myocarditis, epicarditis, and neuroganglionitis, associated with variable focal fibrosis and a paucity of parasites, which is poorly correlated with myocardial inflammatory infiltration [2, 4].

The pathogenesis of CCC includes the balance between parasite invasiveness and the host immune response, particularly the Th1/Th2 balance, which affects the resistance/susceptibility to* T. cruzi* infection [5–7]. Even though an imbalanced, excessive production of Th1 proinflammatory cytokines is critical to control of the parasite levels in blood and tissue [8], this type of response can also be capable of destroying functional cardiomyocytes [8] and intracardiac autonomic neurons [2, 4, 9]. This autonomic denervation, involving mainly parasympathetic postganglionic neurons causes a marked cardiac autonomic dysfunction [2, 4, 9–11], which may be involved in initiating life-threatening cardiac arrhythmias and sudden death [2, 4]. Curiously, this vagal parasympathetic autonomic dysfunction is strongly associated with inflammatory infiltration and immune activation not only in Chagas heart disease but also in other types of cardiopathy [12–15].

Although these data support a direct role of the immune system in mediating both cardiac and autonomic nervous disturbances, recent data indicate that changes in the autonomic nervous system also affect the pattern of immune response and inflammation in several cardiac and noncardiac disease states [15–17], including Chagas disease [18]. Both the sympathetic and parasympathetic branches of the autonomic nervous system can exert substantial modulatory effects on the immune response, mainly by inhibiting the Th1 response profile [17, 19–21]. This new evidence also indicates that cardiac sympathetic and particularly parasympathetic autonomic denervation/dysfunction may also contribute to an increased inflammatory response and possibly to enhanced parasite elimination [22]. Data from our laboratory collected in the context of acute Chagas disease in mice has recently confirmed that cardiac autonomic denervation/dysfunction might contribute to increased inflammation [18].

Taking into account the concepts described above, new therapies based on manipulations of vagal neuroimmunomodulation of the heart may be beneficial for treating heart diseases in general and chronic chagasic cardiomyopathy in particular [17, 23]. For example, therapeutic approaches for increasing cardiac vagal function, thereby potentiating or stimulating the vagal anti-inflammatory reflex, might have a positive impact on chronic chagasic cardiomyopathy. In fact, for other cardiopathies, these strategies, in both an experimental and clinical context, including chronic electric vagal stimulation [24] or pharmacological potentiation [25], have successfully improved cardiac outcomes.

Pyridostigmine bromide, an anticholinesterasic agent that has been used for many years to treat* myasthenia gravis*, exhibits protective cardiovascular effects during short-term administration that lead to a reduction of cardiovascular risk markers and an improvement of autonomic dysfunction [26, 26, 27, 27–29]. By potentiating vagal parasympathetic function, this compound is thought to provoke sinus bradycardia, to reduce AV nodal conduction and the refractory period of action potentials and to increase the cardiac excitation threshold, among other direct cardiac effects. It is also believed that pyridostigmine bromide, via potentiation of the cholinergic anti-inflammatory pathway, causes a reduction in myocardial inflammation and fibrosis associated with an improvement in cardiac hypertrophy and remodeling. However, there have been no reports regarding the effects of pyridostigmine bromide on the chronic phase of Chagas heart disease.

Therefore, the main aim of the present study was to evaluate the effects of cholinergic potentiation with the anticholinesterase agent pyridostigmine bromide on electrocardiographical, cardiac autonomic, histological (inflammatory infiltration and fibrosis), immunological, and parasitological parameters in C57BL/6j mice infected with the Romildo strain of* Trypanosoma cruzi* during the chronic phase of Chagas heart disease.

## 2. Methods

All experiments were performed on wild-type C57BL/6j mice obtained from the animal facility of the Department of Physiology of Triangulo Mineiro Federal University, Uberaba, MG. All animals were males, weighed 20–30 g, and were maintained in the animal facility of the Department of Physiology at the Triangulo Mineiro Federal University on a rodent diet (Nuvilab CR1, Nuvital Nutrientes Ltda, Curitiba, PR, Brazil) and were given water* ad libitum* until the beginning of the experimental protocols. All the experiments were carried out according to the “Principles of Laboratory Animal Care” formulated by the National Society for Medical Research, England, and “The Guide for the Care and Use of Laboratory Animals” published by the US National Institutes of Health (NIH publication number 85-23, revised in 1996). All procedures were also submitted to and approved by the Commission for Ethics in the Use of Animals in Research of the Triangulo Mineiro Federal University.

### 2.1. Parasite Inoculation

To induce experimental Chagas disease, mice were intraperitoneally inoculated with 15,000 trypomastigote forms of the Romildo strain of* T. cruzi*. An additional set of animals matched for gender and weight received intraperitoneal injections of the vehicle, and these were designated as the control noninfected group. After inoculation, all of the animals were observed at least twice daily to monitor their general state and to assess mortality during the acute phase. On the 12th day after inoculation, levels of parasitemia were measured by the microhematocrit method for the peripheral tail blood of all inoculated animals, according to Brenner's technique [30], to confirm infection. All subsequent surgical procedures and experimental protocols were performed at the 5th and 6th months of infection, during the chronic phase of infection, which is characterized by light tissue invasion and low mortality.

### 2.2. Experimental Groups

The experimental groups were divided according to the presence of Chagas disease and the administration of pyridostigmine bromide during the month from the 5th to the 6th month of observation (chronic phase), as described below. The numbers of animals used in each group are indicated in the Results section. The following groups were investigated:

Group I—Con-NT: wild-type C57BL/6j mice were injected with vehicle as a control (Con), not treated (NT) with pyridostigmine bromide and evaluated after six months at the end of the observation period;

Group II—Con-Pyrido: wild-type C57BL/6j were injected with vehicle as a control (Con) and treated with 30 mg/kg pyridostigmine bromide (Pyrido), an anticholinesterase agent, dissolved in tap water, for 30 days from the 5th to the 6th month of observation;

Group III—Chg-NT: wild-type C57BL/6j mice were inoculated with 15,000 trypomastigote forms of the Romildo strain of* T. cruzi* (Chg), not treated (NT) with pyridostigmine bromide and evaluated after six months at the end of the observation period;

Group IV—Chg-Pyrido: wild-type C57BL/6j mice were inoculated with 15,000 trypomastigote forms of the Romildo strain of* T. cruzi* (Chg) and treated with 30 mg/kg pyridostigmine bromide (Pyrido), an anticholinesterase agent, dissolved in tap water, for 30 days from the 5th to the 6th month of observation.

The solution drinking volume was monitored daily, and the pyridostigmine bromide concentration (approximately 0.084 mg/mL) was adjusted daily according to the drinking volume to maintain a daily mean ingested dose of 30 mg/kg.

### 2.3. Conventional Electrocardiographic (ECG) Monitoring in Anesthetized Mice

Immediately prior to inoculation (ECG1), at the 5th (ECG2, immediately prior to the pyridostigmine bromide treatment) and at the 6th months of observation (ECG3, at the end of the experimental protocol, prior to the surgical procedures), all of the animals were submitted to a conventional ECG study under tribromoethanol (250 mg/kg, i.p.) anesthesia. Needle electrodes were placed under the skin to record the conventional bipolar limb leads (I, II, and III), the unipolar limb leads (aVR, aVL, and aVF), and the unipolar precordial (chest) leads (VA: the needle was placed immediately to the right of the sternum in the 4th intercostal space; VB: the needle was placed just to the left of the sternum in the 4th intercostal space; and VC; the needle was positioned in the 5th intercostal space at the midaxillary line). To avoid errors in the positioning of the leads, the same individual consistently placed the electrodes on the animals. The ECG was recorded using an ECG amplifier (model 8811A, Hewlett-Packard Med. Inst., Waltham, MA, USA) coupled to a 12-bit analogue-to-digital interface (DI-720-USB, Dataq Instruments, Inc., Akron, OH, USA) at the sampling rate of 3 KHz on an IBM personal computer with the analysis performed by Windaq-Pro+ software (Dataq Instruments, Inc., Akron, OH, USA). The ECG was recorded for 2 minutes. The intervals and wavelengths (ms) were calculated automatically using customized software following wave identification and cursor placement. The ECG tracings were consistently analyzed by the same individual, who was blinded to the study protocol. The following ECG parameters were examined: (1) RR interval (RRi), (2) P wave duration (Pd), (3) PR interval (PR), (4) QRS duration (QRSd), (5) QT interval (QT), and (6) corrected QT interval (cQT, defined as the QT interval corrected for heart rate using Bazett's equation, where the corrected QTc was equal to QT (in s)/RR (in s)^1/2^). In contrast to humans, the T wave in small rodents is not well characterized and appears as a shoulder of the QRS complex. Accordingly, to measure the QT interval, we used the apex of the T wave, which can be determined with high accuracy. The ECG parameters were determined from each lead and were averaged. Additionally, the presence of cardiac arrhythmias and atrioventricular or intraventricular blockades, among other alterations, was analyzed by visual inspection of ECG tracings by a blinded ECG specialist.

### 2.4. Long-Term Electrocardiographic Recordings in Freely Moving Mice

After the third ECG recording, at the 6th month of observation, the animals were reanesthetized with tribromoethanol (250 mg/kg, i.p.), and a pair of stainless steel electrodes were implanted inside the subcutaneous tissue to collect chronic recordings of conventional bipolar limb ECG lead II. The animals were also cannulated with polyethylene tubing placed in the jugular vein for drug administration. After the surgical procedures, the animals were left to recover in individual cages for at least 48–72 h.

After 48–72 h of surgical recovery and in the absence of anesthesia, the electrodes were connected to an ECG amplifier (model 8811A, Hewlett Packard, Waltham, MA, USA), and the baseline ECG was sampled continuously (3 KHz) for a period of 30 minutes with a personal computer (IBM/PC) equipped with a 12-bit analogue-to-digital interface (DI-720-USB, Dataq Instruments, Inc., Akron, OH, USA). All animals were always recorded between 8:00 AM and 5:00 PM in a quiet condition and in a freely moving state. The time series of RR intervals derived from these chronic ECG recordings were used to study the cardiac autonomic modulation in heart rate variability.

### 2.5. Heart Rate Variability Analysis

From the baseline chronic ECG recordings (30 minutes), the RR interval time series were derived automatically by the detection of the R wave peaks using customized linear analysis software, which was kindly provided by Dr. Alberto Porta (University of Milan, Italy). The time series of the RR intervals were divided into contiguous segments of 300 beats overlapping by half (Welch protocol). After calculating the mean and variance for each segment, a model-based autoregressive spectral analysis was performed, as described elsewhere [31, 32]. Briefly, a model of the oscillatory components present in the stationary segments of the beat-to-beat time series of the RR intervals was calculated based on the Levinson-Durbin recursion, and the order for the model was chosen according to Akaike's criterion [32]. This procedure allows for the automatic quantification of the center frequency and power of each relevant oscillatory component in the time series. The oscillatory components were labeled as very low frequency (VLF), low frequency (LF), or high frequency (HF) when the central frequencies were within the bands of 0.01–0.10 Hz, 0.10–1.00 Hz, or 1.00–5.00 Hz, respectively [18]. The power of the LF and HF components of the heart rate variability was also expressed in normalized units, which were obtained by calculating the percentage of the LF and HF variability with respect to the total power after subtracting the power of the VLF component (frequencies <0.10 Hz). The normalization procedure tends to minimize the effect of changes in the total power on the absolute values of the LF and HF variabilities [31, 32].

### 2.6. Pharmacological Autonomic Blockade

After 30 minutes of baseline chronic ECG recording, half of the animals were given an intravenous injection of atropine sulfate (1 mg/kg, i.v.) followed by an injection of propranolol (1 mg/kg, i.v.) 15 minutes later, whereas the other half received injections in the reverse sequence (propranolol followed by atropine sulfate). This procedure allowed for the quantification of the cardiac parasympathetic and sympathetic autonomic effects, in the former and latter group, respectively, measured as the differences between heart rate (HR) after atropine and baseline HR (parasympathetic effect) or as the differences between HR after propranolol and baseline HR (sympathetic effect), as well as the measurement of intrinsic pacemaker heart rate (IHR), quantified as the heart rate after the double blockade with atropine sulfate followed by propranolol or propranolol followed by atropine sulfate.

### 2.7. Histopathological Examination

At the end of the experimental protocol, all animals were euthanized with an excess dose of sodium thiopental (100 mg/kg, resp., i.p.), and the chest cavity was then opened to remove the heart for histopathological analysis. To evaluate the extent of inflammatory infiltration, tissue damage, fibrosis, and parasite nests, excised hearts from all animals were cleaned in 0.9% saline solution and fixed in phosphate-buffered 10% formalin solution for 48 h.

After embedding the samples in paraffin, five 5–7 *μ*m thick longitudinal (four-chamber) sections of the hearts were stained with hematoxylin-eosin and analyzed using an upright light microscope (Axiolab, Carl Zeiss Inc., Germany). All regions of the hearts were examined by two blinded observers. The inflammatory infiltration and parasite nests were characterized using a semiquantitative approach and the scoring system described by Chapadeiro et al. [33]. A global myocardial inflammation score was defined for each animal as the sum of the scores from different regions of the heart.

To quantify the fibrotic area in the myocardium, contiguous longitudinal sections of the hearts were stained with Picrosirius Red, which binds the collagen present in the tissue matrix, and the slides were analyzed with polarized light microscopy using KS300 software (Karl Zeiss, Inc., Germany).

To enhance the visualization of parasite nests or antigens in cardiac tissue, an immunohistochemistry technique based on the detection of the diaminobenzidine (DAB-) derived chromogen was performed. The chromogen was generated by a secondary antibody labeled with peroxidase, which binds to a primary antibody against* T. cruzi* antigens. After antibody labeling, peroxidase reaction, and costaining with hematoxylin, slides were analyzed with an upright light microscope (Axiolab, Carl Zeiss Inc., Germany).

### 2.8. Quantification of Serum Levels of Cytokines

At the end of the experimental protocol, serum samples for each animal were collected by means of cardiac puncture to perform cytokine profiling (IFN-*γ*, TNF-*α*, IL-2, IL-4, IL-5, and IL-10) via ELISA or the cytometric bead array (CBA) technique.

*Enzyme-Linked Immune Assay (ELISA).* Serum concentrations of IFN-*γ* and IL-10 were measured by ELISA using pairs of monoclonal antibodies in accordance with the manufacturer's specifications (BD Pharmingen). Briefly, high-affinity 96-well plates (Nunc, Roskilde, Denmark) were sensitized with cytokine-specific monoclonal antibodies followed by blocking with PBS containing 2% BSA (Sigma). The sera and recombinant cytokines were then added, and the plates were incubated for 4 h at room temperature. The plates were washed and incubated with 1 *μ*g/mL biotinylated anticytokine monoclonal antibody at 37°C for 2 h followed by washing and incubation with alkaline phosphatase-conjugated streptavidin at 37°C for 2 h. The reaction was developed using disodium-p-nitrophenyl phosphate (Sigma) in diethanolamine buffer. Absorbance was measured at 405 nm in a microplate reader (Bio-Rad, 2550 Reader EIA, CA, USA). The cytokine concentration was calculated using a linear regression analysis of the absorbance values obtained for the recombinant cytokines, and it was expressed as pg/mL. The sensitivity of the tests ranged from 2 to 20 pg/mL.

#### 2.8.1. Flow Cytometry Using a Cytometric Bead Array (CBA)

Measurement of the cytokines TNF-*α* and IL-2 (Th1 cytokines) and IL-4 and IL-5 (Th2 cytokines) in serum samples was performed using the cytometric bead array (CBA) kit (BD Biosciences, USA) according to the manufacturer's instructions. Briefly, 50 *μ*L of bead populations with discrete fluorescence intensities and coated with cytokine-specific capture antibodies was added to 50 *μ*L of mice sera and 50 *μ*L of phycoerythrin-conjugated anti-mouse Th1/Th2 cytokine antibodies. Simultaneously, standards for each cytokine (0–5000 pg/mL) were mixed with cytokine capture beads and the phycoerythrin-conjugated reagent. The vortexed mixtures were incubated for 3 h. Beads were washed and analyzed using flow cytometry (FACSCalibur, BD Biosciences, USA). The quantity (pg/mL) of each cytokine was calculated using CellQuest software (BD Biosciences, USA). Standard curves were derived from the cytokine standards supplied with the kit. The lower limit of detection ranged from 1 to 2.1 pg/mL for different cytokines.

### 2.9. Parasite DNA Detection in the Blood and in the Heart with Conventional PCR

For parasite DNA isolation from the blood, total blood samples of mice were collected in tubes containing 6 M guanidine HCl with 0.2 M EDTA (pH 8) (V/V). DNA was extracted using the phenol-chloroform-isoamyl alcohol method according to Macedo et al. [34].

For parasite DNA isolation from the heart, half of the hearts excised at the end of the experimental protocol were homogenized, and DNA was extracted by the alkaline lysis method.

PCR specific for* T. cruzi* was performed according to Wincker et al. [35] using the following primers to amplify a fragment of 330 bp: 121 (5′-AAA TAA TGT ACG GG(G/T) GAG ATG CAT GA-3′) and 122 (5′-GGT TCG ATT GGG GTT GGT GTA ATA TA-3′) from a nonvariant region of the kinetoplast DNA minicircles of* T. cruzi.* The cycling conditions were as follows: 95°C for 5 minutes followed by 35 cycles at 95°C for 1 minute and 65°C for 1 minute. The products of the reaction were revealed by electrophoresis in a 6.0% polyacrylamide gel and stained with silver nitrate, which was photographed with a digital camera.

### 2.10. Statistical Analysis

All numerical data are expressed as the means (±S.E.M.), whereas the semiquantitative data from the histological examinations are expressed as the medians and the 25th and 75th percentiles. According to the normality and variance homogeneity of the distribution, a parametric statistic, such as two-way ANOVA followed by Tukey's multiple comparison test, or a nonparametric test, such as the Mann-Whitney test, was performed. Categorical variables were analyzed using the Fisher exact test. All statistical calculations were performed using SigmaStat 2.0.3 software (SPSS Inc., Chicago, IL, USA). The differences were considered significant when *P* < 0.05.

## 3. Results

Parasite detection in peripheral tail blood samples by the microhematocrit technique performed on the 12th day after infection was positive for all* T. cruzi-*inoculated animals and negative for all control noninfected mice.

Electrocardiographical parameters measured in the first ECG recording (ECG1) at the beginning of the experimental protocol before* T. cruzi* inoculation and treatment did not show any significant difference among all experimental groups, as expected. However, after five months of infection and before the pyridostigmine bromide treatment, chagasic animals (from Chg-NT and Chg-Pyrido groups) presented significant elongations of Pd, QRSd, QT, and cQT (Table 1), indicating a global functional disturbance of heart.

Interestingly, as shown in Table 2, after one month of the pyridostigmine bromide treatment and six months of infection, Chg-Pyrido mice presented a significant reduction in Pd (an atrial parameter) and cQT (a ventricular parameter), suggesting an improvement in the electrical function of the heart. In contrast, in Chg-NT mice, most of the ECG parameters were significantly different compared to Con-NT mice. It is worth noting that, in the Con-Pyrido group, a significant increase in PR was observed, as expected, because pyridostigmine bromide might be increasing vagal neural transmission in the atrioventricular node, with a consequent reduction in the conduction velocity of the action potential. These results suggest that pyridostigmine bromide treatment can be effective in improving electrical disorders induced by chronic chagasic cardiomyopathy.

The results of heart rate variability analysis in time- (variance) and frequency-domain (spectral) parameters are shown in Table 3. A significant reduction in the absolute values of total variability (variance) and the spectral components VLF, LF, and HF was observed in Chg-NT mice compared to Con-NT and Con-Pyrido mice. The LF and HF components expressed in normalized units and the LF/HF ratio were not different from Con-NT mice. In contrast, chagasic mice treated with pyridostigmine bromide (the Chg-Pyrido group) presented values of variance and VLF and HF spectral components that were significantly higher compared to Chg-NT mice, suggesting an improvement of some autonomic parameters that changed during chronic chagasic cardiomyopathy (Table 3).

**Figure 1:**
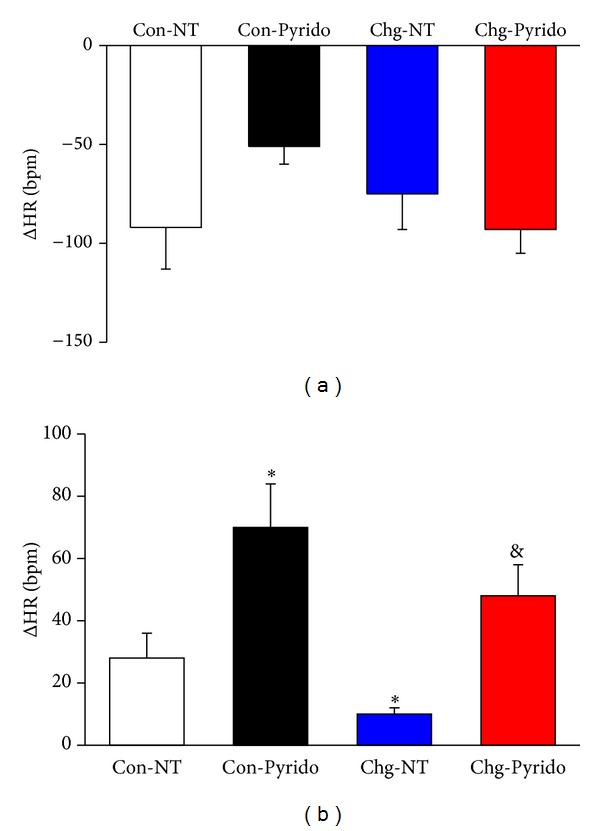
Heart rate responses to pharmacological autonomic blockade. Heart rate responses (**Δ**HR) to propranolol (sympathetic effect) (Panel (a)) or to atropine sulfate (vagal parasympathetic effect) (Panel (b)) expressed as mean ± S.E.M. and measured at the 6th month of observation and after one month of treatment (from the 5th to 6th month of infection) in noninoculated control (Con) or in* T. cruzi-*inoculated (Chg) freely moving C57BL/6j mice treated with pyridostigmine bromide (Pyrido) or vehicle (NT: nontreated animals). (*P < 0.05 versus Con-NT; ^&^ *P* < 0.05 versus Chg-NT).

The autonomic dysfunction observed in the heart rate variability analysis of Chg-NT mice and its improvement in Chg-Pyrido mice was confirmed by pharmacological autonomic blockade with atropine sulfate or propranolol. The use of these autonomic blockers showed that vagal parasympathetic effects significantly decreased without a change in sympathetic effects in Chg-NT mice compared with Con-NT mice (Figure 1). Chagasic mice treated with pyridostigmine bromide showed values of vagal parasympathetic effects that were significantly higher compared to Chg-NT mice and similar to those found in Con-NT mice (Figure 1). Additionally, noninfected control mice treated with pyridostigmine bromide showed vagal parasympathetic effects that markedly increased without any changes in sympathetic effects compared with Con-NT mice (Figure 1). No changes in the intrinsic pacemaker heart rate (IHR) were found in any of the experimental groups after the six-month observation period.

**Figure 2:**
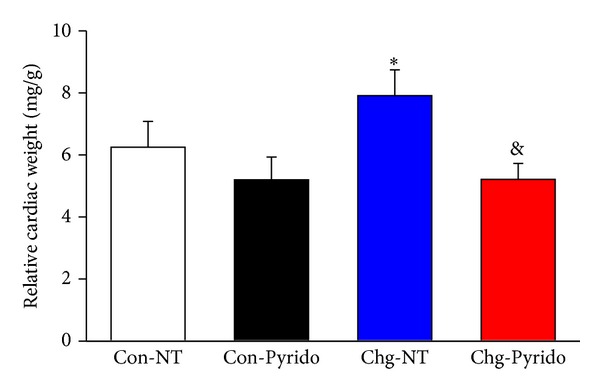
Relative cardiac weights after treatment with pyridostigmine bromide. Relative cardiac weights (in mg/g) expressed as mean ± S.E.M. calculated at the 6th month of observation and after one month of treatment (from the 5th to 6th month of infection) in noninoculated control (Con) or in* T. cruzi-*inoculated (Chg) C57BL/6j mice treated with pyridostigmine bromide (Pyrido) or vehicle (NT: non treated animals). (*P < 0.05 versus Con-NT and ^&^ *P* < 0.05 versus Chg-NT).

The relative cardiac weight of Chg-NT mice was significantly higher than that observed in the other groups (Figure 2), indicating that cardiac hypertrophy was induced by Chagas disease in mice and that pyridostigmine bromide treatment was able to reverse or impair the development of cardiac hypertrophy in chagasic mice.

**Figure 3:**
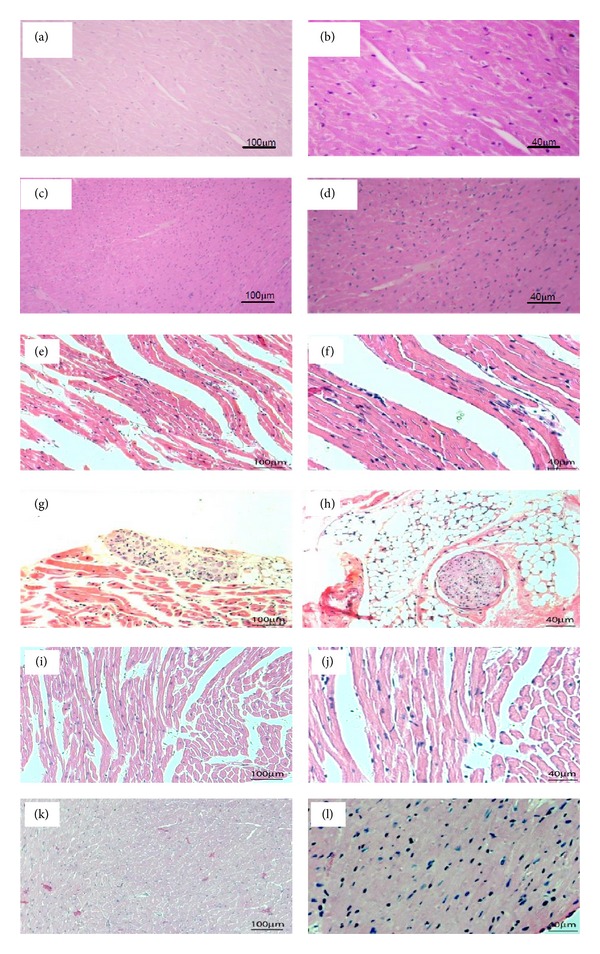
Cardiac histopathology after treatment with pyridostigmine bromide. Photomicrographs of cardiac sections stained with H-E. Panels (a) and (b): ventricular sections from a control nontreated mouse. Panels (c) and (d): ventricular sections from a pyridostigmine bromide-treated control mouse. Panels (e) and (f): ventricular sections from a chagasic nontreated mouse. Panels (g) and (h): atrial sections from a nontreated chagasic mouse showing, respectively, a ganglionitis and neuritis focus. Panels (i), (j), (k), and (l): different ventricular sections from a chagasic mouse treated with pyridostigmine bromide. Note the lower inflammatory infiltration into heart tissues from a chagasic mouse treated with pyridostigmine bromide. (Left panels: magnification = 100x; right panels: magnification = 200x.)

A semiquantitative examination of cardiac inflammatory infiltration indicated discrete to mild diffuse myocarditis (average inflammatory score was equal to 0.5, *P*25% = 0.5, and *P*75% = 0.5, *P* < 0.0001 versus Con-NT), which was observed in the atria (Figures 3(g) and 3(h)), the ventricles (Figures 3(e) and 3(f)), and the neural ganglia (Figure 3(g)) in the CHG-NT mice in comparison to normal CON-NT mice (Figures 3(a), 3(b), 3(c), and 3(d)), which presented no inflammatory infiltration (average inflammatory score was equal to 0.0, *P*25% = 0.0 and *P*75% = 0.0). In Chg-Pyrido mice, reduced inflammatory infiltration, which was categorized as very discrete to discrete (average inflammatory score was equal to 0.125, *P*25% = 0.0, and *P*75% = 0.25, *P* < 0.001 versus Chg-NT), was observed in the atria, the ventricles (Figures 3(i), 3(j), 3(k), and 3(l)), and the neural ganglia. As expected in this mouse model of chronic Chagas disease, no amastigote nests in the myocardium were found in either Chg-NT or Chg-Pyrido mice.

**Figure 4:**
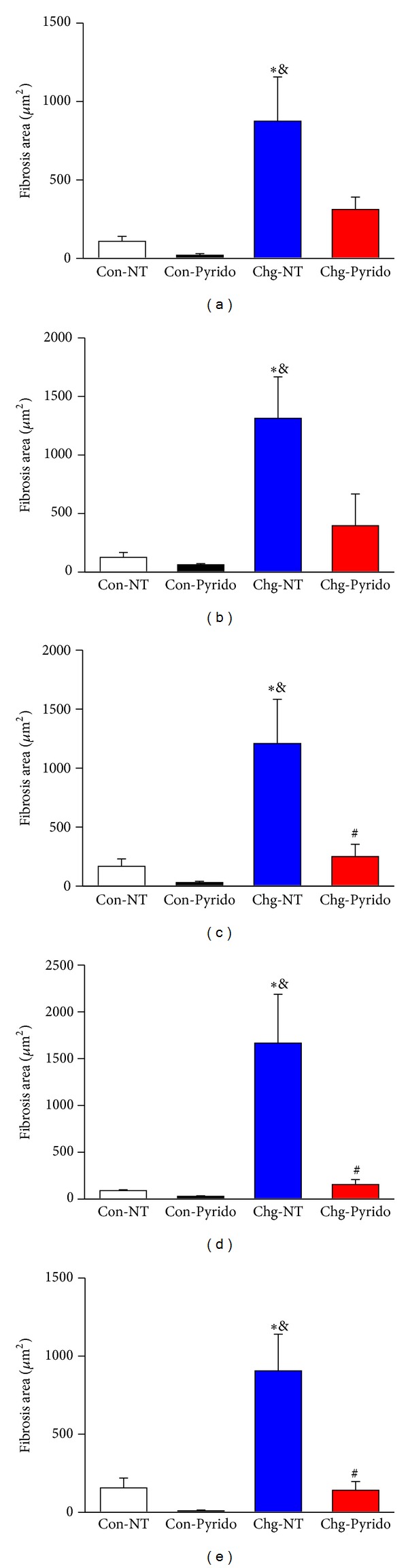
Cardiac fibrosis after treatment with pyridostigmine bromide. Fibrotic areas (in *μ*m^2^) expressed as mean ± S.E.M. for the right (Panel (a)) and left (Panel (b)) atria, the right (Panel (c)) and left (Panel (d)) ventricles, and the atrioventricular septum (Panel (e)), measured after Picrosirius Red staining at the 6th month of observation and after one month of treatment (from the 5th to 6th month of infection) in noninoculated control (Con) or in* T. cruzi-*inoculated (Chg) C57BL/6j mice treated with pyridostigmine bromide (Pyrido) or vehicle (NT: nontreated animals). (*P < 0.05 versus Con-NT; ^&^ *P* < 0.05 versus Chg-Pyrido; and ^#^ *P* < 0.05 versus Con-Pyrido).

In different sections of the heart stained by Picrosirius Red, the morphometry of fibrosis revealed a marked and diffuse increase in the fibrotic area in both absolute (*μ*m^2^) and relative (%) values in the Chg-NT group compared to Chg-NT mice (Figure 4). Interestingly, Chg-Pyrido mice presented significantly reduced fibrotic areas in all cardiac chambers (Figure 4) compared to Chg-NT mice. Despite these reduced values, the fibrotic areas were still larger than those found in Con-NT mice (Figure 4).

**Figure 5:**
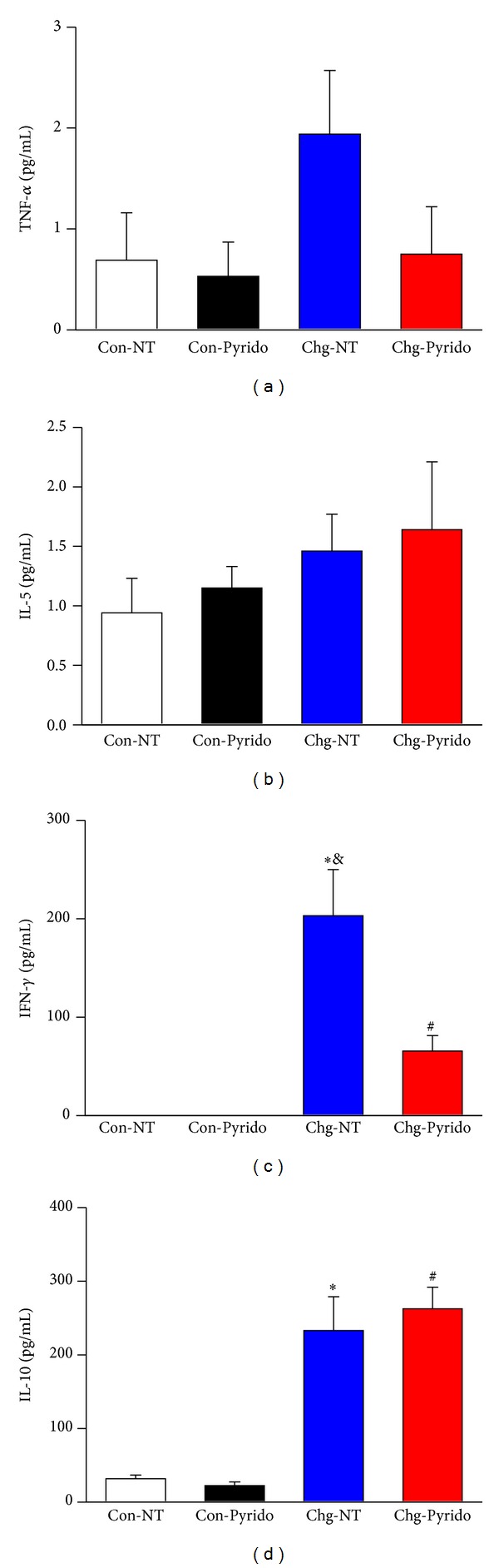
Serum cytokines after treatment with pyridostigmine bromide. Serum levels (in pg/mL) expressed as mean ± S.E.M. of tumor necrosis factor-*α* (TNF-*α*: Panel (a)), interleukin-5 (IL-5: Panel (b)) measured by the cytometric bead array technique, interferon-*γ* (IFN-*γ*: Panel (c)), and interleukin-10 (IL-10: Panel (d)) measured by ELISA at the 6th month of observation and after one month of treatment (from the 5th to 6th month of infection) in noninoculated control (Con) or* T. cruzi-*inoculated (Chg) C57BL/6j mice treated or not (NT) with pyridostigmine bromide (Pyrido). (*P < 0.05 versus Con-NT; ^&^ *P* < 0.05 versus Chg-Pyrido; and ^#^ *P* < 0.05 versus Con-Pyrido).

The serum levels of the cytokines IL-2, IL-4, IL-5, and TNF-*α*, measured with the CBA technique, did not reveal any change in any of the experimental groups. In fact, the serum levels of IL-2 and IL-4 were undetectable, with values near 0 pg/mL. However, using the ELISA technique, serum levels of IFN-*γ* were significantly higher in Chg-NT mice compared to Chg-Pyrido mice (*P* < 0.05). No IFN-*γ* was detected in Con-NT mice. Serum levels of IL-10, measured with ELISA technique, were significantly higher in both Chg-NT and Chg-Pyrido mice compared to Con-NT mice (*P* < 0.05). No differences were found in the serum IL-10 levels between Chg-NT and Chg-Pyrido mice (Figure 5).

**Figure 6:**
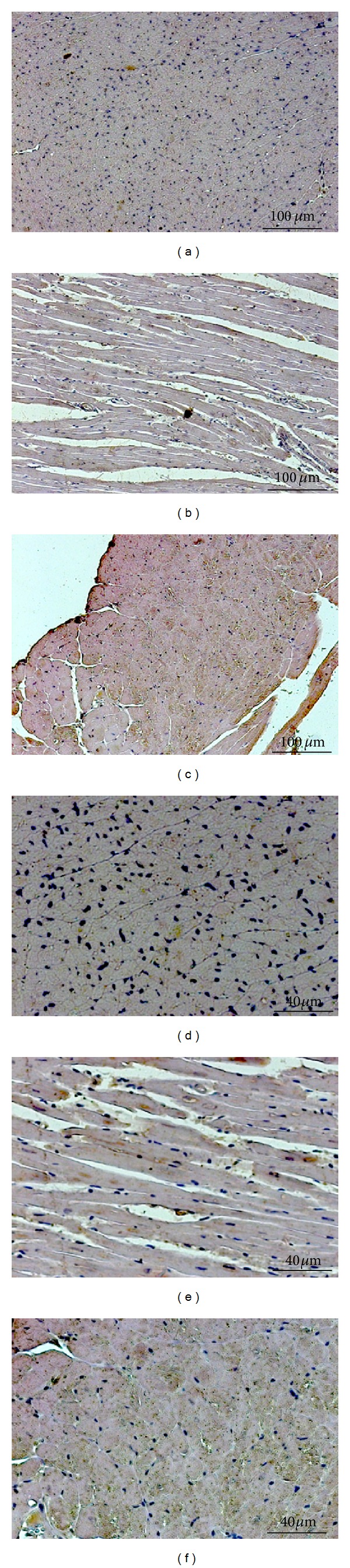
Immunohistochemistry for parasite antigens. Photomicrographs from left ventricular sections stained with* T. cruzi* antigens by peroxidase reaction. Panels (a) and (d): control nontreated mouse. Panels (b) and (e): chagasic nontreated mouse. Panels (c) and (f): chagasic mouse treated with pyridostigmine bromide. Top panels: magnification = 100x; bottom panels: magnification = 200x. Note the gray color labeling* T. cruzi* antigens throughout the sections in Panels (b), (c), (e), and (f).

The immunohistochemical technique using peroxidase to label* T. cruzi* parasite nests or antigens revealed a complete absence of parasites in control noninfected mice, whereas* T. cruzi* labeling was positive for 62.50% of Chg-NT and 81.81% of Chg-Pyrido mice (*P* = 0.603, not significant using the Fisher exact test). The parasite antigens were diffusely distributed throughout the heart tissue (Figure 6).

Using a conventional PCR method to detect parasite DNA in blood and heart during the chronic phase of Chagas disease in mice, a complete absence of parasite DNA in noninfected control mice was observed, whereas* T. cruzi* DNA was found in 16.67% and 42.9% (*P* = 0.559) in the blood of Chg-NT and Chg-Pyrido mice, respectively, and in 72.73% and 86.67% (*P* = 0.614) of heart samples from Chg-NT and Chg-Pyrido mice, respectively.

## 4. Discussion

To our knowledge, the present study is the first to analyze the effects of the deliberate potentiation of cholinergic signaling using pyridostigmine bromide, an anticholinesterase agent, during the chronic phase of experimental Chagas disease in mice. Furthermore, this study evaluated the effects of pyridostigmine bromide treatment on electrocardiographic, autonomic, histopathological, immunoinflammatory, and parasitological parameters of Chagas disease.

The present study is, to our knowledge, the first to demonstrate autonomic dysfunction induced by chronic Chagas disease in an experimental mouse model. This dysfunction was characterized by a marked reduction in heart rate variability, with reduced variance (time-domain parameter) and the VLF, LF, and HF components (frequency-domain parameters) of heart rate variability, as well as a concurrent reduction in the cardiac vagal effect, without an apparent change in sympathetic effects, as measured by pharmacological blockade in chronic control chagasic animals. Only morphological reports describing the ganglia and nerve lesions in chagasic mice have been published in the literature [36]. This finding follows our previous report of a similar autonomic dysfunction in mice with acute Chagas disease [18]. This autonomic dysfunction was accompanied by electrocardiographic changes associated with mild diffuse inflammatory infiltration of mononuclear cells as well as fibrosis and hypertrophy in the atrial and ventricular myocardium and the elevation of IFN-*γ* and IL-10 in the blood serum of wild-type and untreated chagasic C57BL/6j mice, which confirms the presence of chronic myocarditis six months following infection with* T. cruzi*. This increase in serum levels of IFN-*γ* and IL-10 in chronic chagasic mice matches similar previous reports in human beings [37–39]. Additionally, parasite antigens and DNA were detected by immunohistochemical labeling in the heart and by PCR in blood and heart, respectively, reinforcing the role played by parasites in inducing and/or maintaining chronic infection. Because our findings are consistent with previous observations in hamsters, rabbits, dogs, and humans during the chronic phase of Chagas disease [10, 40], the mouse model used here appears to represent a suitable tool for future experimental studies in Chagas heart disease.

During the chronic phase of Chagas heart disease, this report revealed a significant increase in cardiac vagal parasympathetic autonomic modulation associated with a significant reduction of inflammatory infiltration in the myocardium of infected mice treated with pyridostigmine bromide during the last month of a six-month observation period.

Because pyridostigmine bromide is an anticholinesterasic agent, which increases the bioavailability of acetylcholine in the synaptic varicosities, the increase in cardiac vagal parasympathetic autonomic function, demonstrated by the higher heart rate variability and cardiac vagal effect, was expected and was verified after treatment. This vagal stimulation effect of pyridostigmine bromide confirms previous reports in other experimental contexts [41].

The anti-inflammatory effect of pyridostigmine bromide was associated with a marked reduction of myocardial fibrosis and hypertrophy. Serum levels of IFN-*γ* also decreased, whereas TNA-*α* trended to decrease, both of which are Th1 proinflammatory cytokines, without any change in the augmented serum levels of IL-10.

The combined analysis of these results suggests that pyridostigmine bromide may act on the heart, at least partially increasing the vagal parasympathetic activity, via the accumulation of acetylcholine in the myocardium. This accumulation was confirmed by improvements in heart rate variability and vagal parasympathetic effect, which, acting on immune cells, may exert immunomodulatory effects, thereby shifting the immune response toward the predominance of an anti-inflammatory response.

These new findings in the chronic Chagas disease context reinforce the idea of an intimate interplay between the immune and autonomic nervous systems and the potential use of parasympathetic stimulation to treat chagasic myocarditis [18, 23]. Additionally, the improvement of cardiac inflammation, fibrosis, and hypertrophy in pyridostigmine bromide-treated chronic chagasic animals suggests that the decrease of vagal autonomic function due to ganglionic neuronal lesions and denervation, which occur precociously during the acute phase, may play an important immunomodulatory role in the increased Th1 immune response and inflammatory infiltration, as verified during the chronic phase of the disease.

Even though our results support the immunomodulatory role played by the cholinergic vagal parasympathetic nervous system in the heart, we cannot rule out a possible role of pyridostigmine bromide in increasing local acetylcholine levels via its release from nonneural local structures, such as endothelial cells, cholinergic lymphocytes, and cardiomyocytes [42]. In fact, these nonneural sources of acetylcholine, potentiated by pyridostigmine bromide treatment, may explain the substantial improvement in ECG abnormalities, inflammation, fibrosis, and hypertrophy observed in both ventricles of the heart, which are poorly innervated by vagal parasympathetic fibers.

Data from the immunohistochemical labeling of* T. cruzi* antigens and* T. cruzi* DNA detection by PCR showed that parasites are present in the heart tissue and blood of the chagasic mice six months after infection, confirming previous reports [38, 43]. The number of animals positive for parasite antigens or DNA did not differ between pyridostigmine bromide-treated and nontreated chagasic animals. The persistence of the parasite in the chronic phase, even at a very low level, seems to play an important role in the pathogenesis of chronic Chagas disease because the low parasite load may continuously stimulate an immune response [38, 43]. Although antigens or DNA from* T. cruzi* were detected in the present study, no parasite pseudocysts or amastigote forms were observed in the heart tissue in either treated or nontreated chagasic mice. This lack of entire parasites at the site of cardiac lesions suggests the possibility of a remote niche for the parasites, such as smooth muscle cells, adipocytes, or skeletal muscle cells [43].

In conclusion, our results support the notion that autonomic dysfunction is a primary cause of the pro-/anti-inflammatory imbalance observed in inflammatory diseases, favoring the shift of immune response toward the predominance of proinflammatory response, thereby contributing to the pathogenesis of these diseases in general and Chagas heart disease in particular. Additionally, our findings showed the potential beneficial effects of anticholinesterasic agents, particularly pyridostigmine bromide, in increasing cardiac autonomic modulation and reducing inflammatory response to the heart.

## Figures and Tables

**Table 1:** Electrocardiogram before treatment with pyridostigmine bromide. Electrocardiographical parameters (expressed as mean ± S.E.M.) collected after five months follow-up in noninoculated control (Con) or *T. cruzi-*inoculated (Chg) anesthetized C57BL/6j mice before treatment with pyridostigmine bromide (Pyrido) or vehicle (NT: nontreated animals).

	Con-NT	Con-Pyrido	Chg-NT	Chg-Pyrido
	(*n* = 10)	(*n* = 12)	(*n* = 13)	(*n* = 17)
RR (ms)	139.73 ± 5.22	150.83 ± 6.02	139.04 ± 5.62	133.35 ± 4.04
Pd (ms)	13.87 ± 1.50	13.8 ± 1.37	18.56 ± 0.51^∗#^	18.84 ± 0.72^∗#^
PR (ms)	38.83 ± 2.61	38.72 ± 2.28	40.64 ± 0.98	40.17 ± 1.29
QRSd (ms)	14.6 ± 1.86	13.61 ± 1.43	17.53 ± 1.08^∗#^	17.06 ± 1.00^∗#^
QT (ms)	20.74 ± 2.26	17.14 ± 1.60	22.28 ± 0.96^∗#^	21.2 ± 1.01^#^
cQT (ms^1/2^)	1.74 ± 0.51	1.42 ± 0.50	1.9 ± 0.08^∗#^	1.84 ± 0.09^#^

RR: RR interval; Pd: P wave duration; PR: PR interval; QRSd: QRS complex duration; QT: QT interval; cQT: corrected QT interval; ms: milliseconds; *P < 0.05 versus Con-NT; and ^#^ *P* < 0.05 versus Con-Pyrido.

**Table 2:** Electrocardiogram after treatment with pyridostigmine bromide. Electrocardiographical parameters (expressed as mean ± S.E.M.) collected at the 6th month of observation and after one month of treatment (from the 5th to 6th month of infection) in noninoculated control (Con) or *T. cruzi-*inoculated (Chg) anesthetized C57BL/6j mice treated with pyridostigmine bromide (Pyrido) or vehicle (NT: nontreated animals).

	Con-NT	Con-Pyrido	Chg-NT	Chg-Pyrido
	(*n* = 10)	(*n* = 12)	(*n* = 10)	(*n* = 17)
RR (ms)	137.56 ± 3.37	141.05 ± 5.38	152.54 ± 5.01∗	152.98 ± 12.93
Pd (ms)	14.01 ± 1.53	13.38 ± 1.51	18.68 ± 0.52^∗&#^	14.63 ± 0.53
PR (ms)	37.93 ± 1.96	44.38 ± 2.51∗	45.45 ± 2.22∗	43.2 ± 0.99
QRSd (ms)	14.45 ± 1.39	13.47 ± 1.37	15.04 ± 0.53	14.5 ± 0.41
QT (ms)	17.77 ± 1.87	17.35 ± 1.38	22.47 ± 1.04^∗#^	18.21 ± 0.59
cQT (ms^1/2^)	1.51 ± 0.51	1.49 ± 0.47	1.83 ± 0.09^∗&#^	1.51 ± 0.06

RR: RR interval; Pd: P wave duration; PR: PR interval; QRSd: QRS complex duration; QT: QT interval; cQT: corrected QT interval; *P < 0.05 versus Con-NT; ms: milliseconds; ^&^ *P* < 0.05 versus Chg-Pyrido; and ^#^ *P* < 0.05 versus Con-Pyrido.

**Table 3:** Heart rate variability after treatment with pyridostigmine bromide. Heart rate variability parameters (expressed as mean ± S.E.M.) collected at the 6th month of observation and after one month of treatment (from the 5th to 6th month of infection) in noninoculated control (Con) or *T. cruzi-*inoculated (Chg) freely moving C57BL/6j mice treated with pyridostigmine bromide (Pyrido) or vehicle (NT: nontreated animals).

	Con-NT	Con-Pyrido	Chg-NT	Chg-Pyrido
	(*n* = 9)	(*n* = 9)	(*n* = 9)	(*n* = 12)
RR (ms)	117.97 ± 3.97	112.67 ± 4.14	104.27 ± 4.32	113.74 ± 4.33
HR (bpm)	516.81 ± 8.35	543.57 ± 9.06	590.61 ± 9.82	539.07 ± 8.76
Variance (ms^2^)	218.61 ± 12.35	148.96 ± 9.91	32.13 ± 5.12^∗&^	63.57 ± 6.11
VLF (ms^2^)	106.06 ± 8.93	69.37 ± 7.23	11.76 ± 2.84^∗&^	22.60 ± 3.90
LF (ms^2^)	57.84 ± 6.28	52.31 ± 6.23	11.10 ± 3.77∗	21.99 ± 5.09
LF (nu)	51.83 ± 4.13	56.24 ± 4.09	47.80 ± 4.57	39.25 ± 4.91
HF (ms^2^)	54.71 ± 6.86	27.19 ± 4.44	9.01 ± 3.19^∗&^	18.78 ± 3.53
HF (nu)	47.54 ± 4.11	41.80 ± 3.97	48.05 ± 4.49	58.70 ± 4.86
LF/HF	2.31 ± 1.53	4.04 ± 1.48	2.60 ± 1.54	2.92 ± 2.01

RR: RR interval; HR: heart rate; VLF: very low frequency spectral component; LF: low frequency spectral component; HF: high frequency spectral component; ms: milliseconds; bpm: beats per minute; nu: normalized units; *P < 0.05 versus Con-NT; and ^&^ *P* < 0.05 versus Chg-Pyrido.

## References

[1] Chagas CJ (1911). American trypanosomiasis. *Memórias do Instituto Oswaldo Cruz*.

[2] Prata A (2001). Clinical and epidemiological aspects of Chagas disease. *The Lancet Infectious Diseases*.

[3] Coura JR, Vĩas PA (2010). Chagas disease: a new worldwide challenge. *Nature*.

[4] Marin-Neto JA, Cunha-Neto E, Maciel BC, Simões MV (2007). Pathogenesis of chronic Chagas heart disease. *Circulation*.

[5] Morris SA, Tanowitz HB, Wittner M, Bilezikian JP (1990). Pathophysiological insights into the cardiomyopathy of Chagas’ disease. *Circulation*.

[6] Alves MJM, Mortara RA (2009). A century of research: What have we learned about the interaction of *Trypanosoma cruzi* with host cells?. *Memorias do Instituto Oswaldo Cruz*.

[7] Hunter CA, Ellis-Neyes LA, Slifer T (1997). IL-10 is required to prevent immune hyperactivity during infection with *Trypanosoma cruzi*. *The Journal of Immunology*.

[8] Teixeira ARL, Hecht MM, Guimaro MC, Sousa AO, Nitz N (2011). Pathogenesis of Chagas’ disease: parasite persistence and autoimmunity. *Clinical Microbiology Reviews*.

[9] Ramirez LE, Lages-Silva E, Soares JM, Chapadeiro E (1993). Experimental hamster infection by *Trypanosoma cruzi*: the chronic phase. *Revista da Sociedade Brásileira de Medicina Tropical*.

[10] da Silva VJD, Machado MPR, Rocha AM, Padilha RM, Ramirez LE (2003). Analysis of cardiac autonomic function in hamsters with Chagas disease. *Revista da Sociedade Brasileira de Medicina Tropical*.

[11] Pérez AR, Silva-Barbosa SD, Berbert LR (2011). Immunoneuroendocrine alterations in patients with progressive forms of chronic Chagas disease. *Journal of Neuroimmunology*.

[12] Kleiger RE, Miller JP, Bigger JT, Moss AJ (1987). Decreased heart rate variability and its association with increased mortality after acute myocardial infarction. *The American Journal of Cardiology*.

[13] Hamaad A, Sosin M, Blann AD, Patel J, Lip GYH, MacFadyen RJ (2005). Markers of inflammation in acute coronary syndromes: association with increased heart rate and reductions in heart rate variability. *Clinical Cardiology*.

[14] Hauptman PJ, Schwartz PJ, Gold MR (2012). Rationale and study design of the INcrease of Vagal TonE in Heart Failure study: INOVATE-HF. *The American Heart Journal*.

[15] Papaioannou V, Pneumatikos I, Maglaveras N (2013). Association of heart rate variability and inflammatory response in patients with cardiovascular diseases: current strengths and limitations. *Front Physiol*.

[16] van Westerloo DJ, Giebelen IAJ, Florquin S (2005). The cholinergic anti-inflammatory pathway regulates the host response during septic peritonitis. *Journal of Infectious Diseases*.

[17] Tracey KJ (2009). Reflex control of immunity. *Nature Reviews Immunology*.

[18] Machado MPR, Rocha AM, de Oliveira LF (2012). Autonomic nervous system modulation affects the inflammatory immune response in mice with acute Chagas disease. *Experimental Physiology*.

[19] Sanders VM (1998). The role of norepinephrine and beta-2-adrenergic receptor stimulation in the modulation of TH1, TH2, and B lymphocyte function. *Advances in Experimental Medicine and Biology*.

[20] Elenkov IJ, Chrousos GP (1999). Stress hormones, Th1/Th2 patterns, pro/anti-inflammatory cytokines and susceptibility to disease. *Trends in Endocrinology and Metabolism*.

[21] Borovikova LV, Ivanova S, Zhang M (2000). Vagus nerve stimulation attenuates the systemic inflammatory response to endotoxin. *Nature*.

[22] Benarroch EE (2009). Autonomic-mediated immunomodulation and potential clinical relevance. *Neurology*.

[23] Machado MPR, da Silva VJD (2012). Autonomic neuroimmunomodulation in chagasic cardiomyopathy. *Experimental Physiology*.

[24] Li M, Zheng C, Sato T, Kawada T, Sugimachi M, Sunagawa K (2004). Vagal nerve stimulation markedly improves long-term survival after chronic heart failure in rats. *Circulation*.

[25] Okazaki Y, Zheng C, Li M, Sugimachi M (2010). Effect of the cholinesterase inhibitor donepezil on cardiac remodeling and autonomic balance in rats with heart failure. *The Journal of Physiological Sciences*.

[26] Serra SM, Costa RV, Teixeira de Castro RR, Xavier SS, Lucas da Nóbrega AC (2009). Cholinergic stimulation improves autonomic and hemodynamic profile during dynamic exercise in patients with heart failure. *Journal of Cardiac Failure*.

[27] Zimerman LI, Liberman A, Castro RRT, Ribeiro JP, Nóbrega ACL (2010). Acute electrophysiologic consequences of pyridostigmine inhibition of cholinesterase in humans. *Brazilian Journal of Medical and Biological Research*.

[28] Castro RRT, Porphirio G, Serra SM, Nóbrega ACL (2004). Cholinergic stimulation with pyridostigmine protects against exercise induced myocardial ischaemia. *Heart*.

[29] Castro RRT, Serra SM, Porphirio G, Mendes FSNS, Oliveira LPJ, Nóbrega ACL (2006). Pyridostigmine reduces QTc interval during recovery from maximal exercise in ischemic heart disease. *International Journal of Cardiology*.

[30] Brener Z (1962). Therapeutic activity and criterion of cure on mice experimentally infected with Trypanosoma cruzi. *Revista do Instituto de Medicina Tropical de São Paulo*.

[31] Task Force of the European Society of Cardiology and the North American Society of Pacing and Electrophysiology (1996). Heart rate variability: standards of measurement, physiological interpretation and clinical use. *Circulation*.

[32] Montano N, Porta A, Cogliati C (2009). Heart rate variability explored in the frequency domain: a tool to investigate the link between heart and behavior. *Neuroscience and Biobehavioral Reviews*.

[33] Chapadeiro E, Beraldo PS, Jesus PC, Oliveira Júnior WP, Junqueira Júnior LF (1988). Cardiac lesions in Wistar rats inoculated with various strains of *Trypanosoma cruzi*. *Revista da Sociedade Brasileira de Medicina Tropical*.

[34] Macedo AM, Martins MS, Chiari E, Pena SDJ (1992). DNA fingerprinting of *Trypanosoma cruzi*: a new tool for characterization of strains and clones. *Molecular and Biochemical Parasitology*.

[35] Wincker P, Bosseno M, Britto C (1994). High correlation between Chagas' disease serology and PCR-based detection of *Trypanosoma cruzi* kinetoplast DNA in Bolivian children living in an endemic area. *FEMS Microbiology Letters*.

[36] Ribeiro LCV, Barbosa AA, Andrade ZA (2002). Pathology of intracardiac nerves in experimental chagas disease. *Memorias do Instituto Oswaldo Cruz*.

[37] Ferreira RC, Ianni BM, Abel LCJ (2003). Increased plasma levels of tumor necrosis factor-alpha in asymptomatic/“indeterminate” and Chagas disease cardiomyopathy patients. *Memorias do Instituto Oswaldo Cruz*.

[38] Gutierrez FRS, Guedes PMM, Gazzinelli RT, Silva JS (2009). The role of parasite persistence in pathogenesis of chagas heart disease. *Parasite Immunology*.

[39] de Melo AS, de Lorena VMB, de Moura Braz SC, Docena C, de Miranda Gomes Y (2012). IL-10 and IFN-*γ* gene expression in chronic Chagas disease patients after in vitro stimulation with recombinant antigens of *Trypanosoma cruzi*. *Cytokine*.

[40] Soares MB, dos Santos RR (1999). Immunopathology of cardiomyopathy in the experimental Chagas disease. *Memórias do Instituto Oswaldo Cruz*.

[41] de la Fuente RN, Rodrigues B, Moraes-Silva IC (2013). Cholinergic stimulation with pyridostigmine improves autonomic function in infarcted rats. *Clinical and Experimental Pharmacology and Physiology*.

[42] Rocha-Resende C, Roy A, Resende R (2012). Non-neuronal cholinergic machinery present in cardiomyocytes offsets hypertrophic signals. *Journal of Molecular and Cellular Cardiology*.

[43] Nagajyothi F, Machado FS, Burleigh BA (2012). Mechanisms of Trypanosoma cruzi persistence in Chagas disease. *Cellular Microbiology*.

